# Promoting trust in research and researchers: How open science and research integrity are intertwined

**DOI:** 10.1186/s13104-022-06169-y

**Published:** 2022-09-20

**Authors:** Tamarinde Haven, Gowri Gopalakrishna, Joeri Tijdink, Dorien van der Schot, Lex Bouter

**Affiliations:** 1 BIH QUEST Center for Responsible Research, Charité Universitätsmedizin, Berlin, Germany; 2 Department of Epidemiology and Data Science, Amsterdam University Medical Centers, Amsterdam, The Netherlands; 3 Department of Ethics, Law and Humanities, Amsterdam University Medical Centers, Amsterdam, the Netherlands; 4 Department of Philosophy, Faculty of Humanities, Vrije Universiteit, Amsterdam, Netherlands

**Keywords:** Open science, Research integrity, Transparency, Responsible research practices

## Abstract

Proponents of open science often refer to issues pertaining to research integrity and vice versa. In this commentary, we argue that concepts such as responsible research practices, transparency, and open science are connected to one another, but that they each have a different focus. We argue that responsible research practices focus more on the rigorous *conduct* of research, transparency focuses predominantly on the complete *reporting* of research, and open science’s core focus is mostly about *dissemination* of research*.* Doing justice to these concepts requires action from researchers and research institutions to make research with integrity possible, easy, normative, and rewarding. For each of these levels from the Center for Open Science pyramid of behaviour change, we provide suggestions on what researchers and research institutions can do to promote a culture of research integrity. We close with a brief reflection on initiatives by other research communities and stakeholders and make a call to those working in the fields of research integrity and open science to pay closer attention to one other’s work.

## Introduction

Highly publicised cases of research misconduct [1] have led to negative attention towards research integrity in which falsification, fabrication, and plagiarism are considered the three ‘cardinal sins’ of a researcher. Similarly, concerns about reproducibility [2–4]^1^ triggered debates about the extent to which research is in an alleged crisis. At the same time, there is an increasing push to make research more open, which tends to carry with it more positive connotations. Open science has increasingly become a topic that is discussed and appreciated across different disciplines. Some funding agencies and scholarly journals recently started to mandate open science practices such as making research data public [5].

Despite these different connotations, proponents of Open Science refer to issues pertaining to research integrity and vice versa [6]. In this commentary, we show how some of the frequently used concepts (research integrity, responsible research practices, transparency, and open science) interrelate. The upshot of our commentary is that these concepts are all crucial to strengthen trust in research and researchers by making research more traceable and verifiable. We believe that their focus on a particular phase of the research process can be instrumental to further the understanding of these concepts, because it is precisely by virtue of their different foci that these concepts become complementary and mutually reinforcing. We illustrate this using an example of an imaginary research project, and by providing examples of situations where one concept is missing. We then elaborate on the different factors that influence research integrity and connect this to what research institutions can do to foster research integrity.

## Main text

### Intertwining concepts

**Fig. 1:**
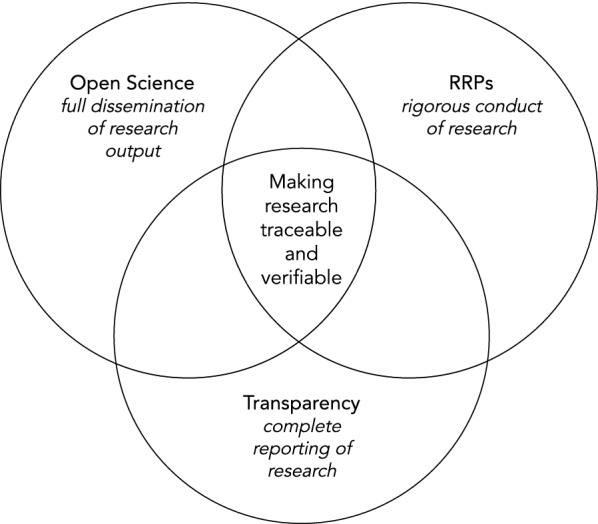
Intertwined concepts of responsible research practices, transparency, open science and their foci. This Venn diagram illustrates how the different concepts interrelate to make research more traceable and verifiable, with the aim to increase trust in research and researchers

In a nutshell, we believe that responsible research practices focus more on the rigorous *conduct of research*, transparency focuses predominantly on the complete *reporting of research* at every stage of the research lifecycle, and open science’s core focus is mostly on the *dissemination of research*, see Fig. 1. Having said that, we wish to emphasise at this point to the reader that we are not suggesting these concepts are mutually exclusive which implies that in a number of instances, the concepts can be, and in fact are, used interchangeably.

Research integrity goes well beyond research misconduct (i.e., fabrication—making up of data that does not exist, falsification—manipulating data or results without justification, or plagiarism (FFP) [7]. It refers to the “principles and standards that have the purpose to ensure validity and trustworthiness of research” [8]. Research integrity focuses on the behaviour of individual researchers which is often grouped into three clusters: FFP, questionable research practices (QRPs), and responsible research practices (RRPs) [9]. FFP is obviously detrimental to trust in research, but we have some reason to believe it is relatively rare [10]. QRPs, which entail behaviours such as selectively reporting, p-hacking, or HARK-ing (hypothesising after results are known) are thought to be more common and to do collectively more damage than FFP [10, 11]. Although QRPs may be the result of sloppiness, engaging in these behaviours may also be intentional with the aim of having clean, clear-cut research findings in the hope of getting it published in a prestigious journal. RRPs are behaviours a researcher can engage in that can help ensure the quality and trustworthiness of one’s research. What these behaviours have in common, is that they focus on the way research is *conducted*. Examples include applying validated measurement instruments, consulting with a statistician regarding the appropriateness of the proposed data analysis models, keeping a comprehensive record of the decisions made during the research process and meticulously checking a manuscript to avoid errors [12].

Transparency comes into play when *reporting* how a study was or will be conducted, for example, by writing up a detailed research protocol before the start of the study and reporting all its results afterwards. Additional examples include open notebooks or open lab books [13], where researchers make the entire process of their research available, not just their protocols or final results. Here, a high level of detail is important. This detailed insight enables the readers or reviewers of the manuscript to draw their own conclusions about the credibility of the findings because they get a complete insight into how the study was set up or conducted.

Open science is an umbrella term. When it comes to what researchers can do to make their work more traceable and verifiable, a major part of open science focuses on how research output is *disseminated* [14–16]. Open science has also broadened the traditional interpretation of what counts as research output: proponents of open science plead for publicly sharing study methods via preregistrations (e.g., via osf.io), depositing or publishing study protocols and data analysis plans in a relevant repository (e.g., protocols.io or plos.org/protocols), publicly sharing code used to analyse the data, the complete data set in itself plus its metadata, and making the study findings rapidly and freely available as a preprint to be, ideally, followed by an open peer-reviewed open access publication.

Let’s review an example research project to see how these concepts strengthen one other. A research team is interested in the effect of Covid-19 restrictions on adolescents. One team member identifies if there are validated, target population-oriented questionnaires available that are relevant for answering the research question of interest (*conduct of research*). Another team member calculates the appropriate sample size for detecting the effect size of interest (*conduct of research).* The team then incorporate this information and start drafting a study protocol. They preregister this protocol in a publicly accessible repository that allows reviewers or colleagues to assess whether they have done what they promised to do (*reporting of research).* The team proceeds with data collection and analysis. They use a reporting guideline to structure writing up their findings and to assure relevant details are included the in the manuscript (*reporting of research)*. Because the team wants to practice open science, they publish their data in a way that assures it is Findable, Accessible, Interoperable and Reusable (FAIR [17] *dissemination of research*). However, to assure that the data is useful to others, the team devotes great care to describing a comprehensive code book that is linked to their dataset, explaining the metadata and variable names (*reporting of research*).

Now let’s review an example where research that is open and transparent, but not rigorous. A study can be preregistered with its full study protocol and share its data in a FAIR format, but if those data have been collected using sloppy methods (e.g., the study was not randomized, had a low sample size, was non-blinded, reported irrelevant outcomes or assessed relevant outcomes poorly, etc.), it still is a poor-quality study. Here open science enables greater transparency, allowing others to assess the protocol, method, data, analysis, and conclusions to determine if the study is rigorously done and bias is avoided. In this way we can assess research quality because all parts of the research are open for scrutiny by others.

A study can also have applied rigorous methods and be transparently reported, but if it is not openly accessible, it will only be read and used by a subset of the target community. Most researchers know some way around paywalls, but it could be missed by relevant policy makers, leading to potentially distorted policies or guidelines.

A study can also be rigorously conducted and openly accessible, but if it is not transparently reported, there is a risk that readers do not fully understand the data collection and analysis methods applied. This could lead to flawed interpretations, or perhaps to disregarding a useful study altogether because its credibility is believed to be low.

These examples serve to highlight some of the ways in which RRPs, transparency and open science reinforce one another. RRPs aid in lowering the risk of bias and strengthen the study quality. Open science facilitates transparency by providing the infrastructure for sharing study details (without space, word or paywall limitations). Some open science formats such as preregistration may help detect QRPs such as selective reporting and data-driven modifications of the research protocol and the data-analysis plan since it offers readers full and open access to all study details determined a priori. Transparency on the approach taken and the data generated are crucial for open science practices to be meaningful. Transparent reporting also makes it possible, if necessary, to carry out a replication study [18] or to reuse the data for a pooled data synthesis or to answer other research questions. By doing so, trust in research and researchers may be (justifiably) strengthened.

### What can researchers and research institutions do?

The actions described above are ultimately in the hands of individual researchers. But what drives researchers to conduct their work with integrity? Building on the Center for Open Science pyramid of behaviour change [19], we review what researchers and research institutions can do to promote a culture of research integrity.

To make conducting research with integrity *possible*, it is essential that research institutions have the necessary infrastructure to curate and store data in accordance with FAIR principles.

To make it *easy*, research institutions should have the right support for researchers. This could range from research data management experts to statisticians. It also means that the systems researchers need to work with to enable long-term storage of data or to request statistical support are user-friendly. In addition, institutions can support their researchers by providing state of the art research integrity training [20]. This training can also be done more informally through community efforts (e.g., ReproducibiliTea (reproducibilitea.org) or Open Science Communities (openscience.nl)). A combination of formally integrated into curriculum or professional development education and more informal initiatives is probably the quickest and most efficient way to achieve a change in culture. In addition, institutions can support their faculty tasked with mentoring more junior researchers with training in performing these responsibilities [21].

To make it *normative*, individual researchers can further promote cultural change by role modelling rigorous conduct of research or by teaming up in grassroots initiatives that lobby for change at the institutional level (e.g., the United Kingdom Reproducibility Network, ukrn.org). Factors like mentoring for survival (i.e., socialising early career researchers into the ‘art’ of cutting corners with a view to maximize the number of publications, citations, and grants [22]) may undermine research integrity. On the other hand, adherence to scientific norms such as assessing validity based on the research not the researcher and critically appraising research findings before accepting them (also known as Mertonian norms [23]) was shown to reduce the likelihood of QRPs, and research misconduct while promoting RRPs [12]. Other factors like responsible mentoring could also promote research integrity [24].

To make it *rewarding*, research institutions should pay due attention to reward their researchers for conducting their work with integrity. It has become apparent that if researchers feel treated unfairly by their department or institution, they may be more likely to engage in questionable research practices, or worse, to compensate for this perceived unfairness [12]. Research institutions should have policies and procedures in place to fairly assess researchers [25] and ensure that research integrity is embedded into those policies [26].

Other perverse incentives also play an important role, such as the quantification and commodification of research [27]. This results in assessment systems that makes funding, quantitative scientific output, and number of students important parameters in the financial resources available to universities [28]. There are various international efforts to change researcher assessment systems. One of the most visible is San Francisco Declaration on Research Assessment (DORA, see sfdora.org). A substantial number of research institutions have signed DORA and are committed to implement the DORA recommendations into their internal criteria for promotion, meaning that they also reward researchers that make their work openly and transparently available. More specifically, the Hong Kong Principles [29] outline how the assessment of researchers can be reformed to foster research integrity and open science practices.

### Outlook

We have discussed concepts mainly with empirical quantitative research in mind. It is worth noting, however, that there are interesting initiatives going on in parts of the humanities [30]. For example, researchers are trying to perform a replication study in the field of history [31]. In addition, there is discussion about preregistering some forms of qualitative research [32].

Whereas we focused on what individual researchers and research institutions can do to promote the quality and trustworthiness of research, it is fair to say that other stakeholders like journals and funders play an equally important role, particularly in shaping factors at the more systemic level. Ultimately improving trust in research and researchers will require concerted efforts from all stakeholders.

Our take home message is that researchers interested in open science should pay attention to the work of their peers in the adjacent community of research integrity, and vice versa. Both communities are growing and in order to prevent duplicate efforts, it is crucial to keep track of others’ work, and to collaborate more closely in promoting trust in research and researchers.

## Data Availability

Not applicable.

## Abbreviations

FFP: Falsification, fabrication, and plagiarism
QRPs: Questionable Research Practices
RRPs: Responsible Research Practices
FAIR: Findable, Accessible, Interoperable, Reusable

## Footnotes

1 Reproducibility and replicability are often used interchangeably, here we use the formulation based on the report by the National Academies (3), but other conceptualisations exist, too (4).
